# Estimation of effective half-life and lesion-specific kinetics in I-131 therapy for differentiated thyroid cancer using dual-timepoint imaging

**DOI:** 10.1186/s40658-026-00916-x

**Published:** 2026-07-07

**Authors:** Yusuke Iizuka, Ryota Nakashima, Rihito Aizawa, Masaaki Kajisako, Maki Shioji, Katsuhiko Mitsumoto, Koji Itagaki, Shigeto Kawase, Takashi Mizowaki

**Affiliations:** 1 Department of Radiation Oncology and Image-Applied Therapy, Graduate School of Medicine, Kyoto University, 54 Shogoin-Kawaharacho, Sakyo-ku, Kyoto, 6068507 Japan; 2 Division of Clinical Radiology Service, Kyoto University Hospital, Kyoto, Japan

**Keywords:** Effective half-life, I-131 therapy, Differentiated thyroid cancer, Dual-timepoint imaging, Patient-specific dosimetry, Quantitative imaging

## Abstract

**Background:**

Accurate estimation of lesion-specific effective half-life (T_eff_) is essential for patient-specific dosimetry in radioiodine (I-131) therapy for differentiated thyroid cancer (DTC). Although conventional multi-timepoint imaging provides detailed kinetic information, it is often impractical for routine clinical implementation. This study prospectively evaluated a simplified dual-timepoint planar imaging protocol aimed at balancing quantitative reliability with clinical feasibility.

**Methods:**

Fifty patients with DTC undergoing I-131 therapy were prospectively analyzed. Whole-body planar images were acquired twice after administration: an early scan at 1.98–3.25 days (mean, 2.71 days) and a delayed scan at 8.97–17.16 days (interval between the two scans ranged from 6.18 to 14.28 days [mean, 12.35 days]). Lesion-specific T_eff_ values were calculated assuming mono-exponential clearance between the two scans using a log-linear approximation of measured activity counts. Quantitative reproducibility was assessed by comparing activity–count calibration slopes derived from an internal reference I-131 capsule between independent imaging sessions.

**Results:**

The mean T_eff_ was 2.16 ± 0.56 days and 3.11 ± 0.74 days for thyroid bed (*n* = 35) and metastatic (*n* = 19; *p* < 0.001) lesions, respectively. Among metastatic sites, bone and lung metastases showed numerically similar T_eff_ values (3.22 ± 0.76 and 3.36 ± 0.80 days, respectively); however, this subgroup comparison was limited by the small sample size. Calibration slopes derived from the internal reference capsule demonstrated only 5.0% inter-session variability, indicating high quantitative reproducibility.

**Conclusions:**

Dual-timepoint planar imaging incorporating internal capsule calibration enables reproducible estimation of lesion-specific T_eff_ in patients undergoing I-131 therapy for DTC. This approach achieves a practical balance between quantitative accuracy and clinical feasibility, enabling reliable characterization of iodine kinetics with minimal imaging burden and supporting individualized dosimetry in routine clinical practice.

## Background

Differentiated thyroid cancer (DTC) is the most common endocrine malignancy and generally carries an excellent prognosis when treated appropriately [1, 2]. Radioiodine (I-131) therapy plays an important role in the postoperative management of remnant ablation, adjuvant treatment, and management of metastatic disease [3]. Despite its long-standing clinical use, the administered activity of I-131 is still commonly determined empirically according to risk stratification, rather than based on patient- or lesion-specific dosimetric considerations.

Internal dosimetry has the potential for optimizing I-131 therapy by individualizing administered activity according to the absorbed dose. A key parameter in internal dosimetry is the effective half-life (T_eff_), which reflects both the physical decay of I-131 and its biological clearance from tissues [4, 5]. Accurate estimation of lesion-specific T_eff_ is therefore essential for reliable absorbed dose calculation. However, conventional approaches for estimating T_eff_ rely on serial imaging at multiple time points for constructing time–activity curves, which is logistically demanding and challenging to implement in routine clinical practice.

To improve feasibility, simplified dosimetric approaches using fewer imaging time points have been proposed. Among these, dual-timepoint imaging represents a practical compromise, provided that iodine clearance follows a predominantly mono-exponential pattern after the early uptake phase. Previous studies have suggested that T_eff_ and absorbed dose estimates derived from limited timepoint data can remain within acceptable error ranges for clinical application [6]. Nevertheless, the quantitative reliability and reproducibility of such simplified methods depend critically on imaging stability, calibration accuracy, and accurate timing of image acquisition.

Quantitative imaging of I-131 presents specific technical challenges owing to its high-energy gamma emissions, septal penetration, and scatter, which can compromise image-based activity estimation. Although single-photon emission computed tomography (SPECT)/computed tomography (CT) offers theoretical advantages for three-dimensional quantification, reconstruction-dependent factors and high-count artifacts may adversely affect quantitative stability. In our previous study, we demonstrated that planar imaging with an internal reference I-131 capsule provided more robust and reproducible activity quantification than SPECT imaging under clinical conditions [7]. These findings suggest that planar imaging may offer advantages for stable dosimetric measurements, particularly when combined with internal calibration.

Building on this prior work, the present study aimed to evaluate whether dual-timepoint planar imaging with internal capsule calibration can provide reproducible and clinically meaningful estimates of lesion-specific T_eff_ in patients undergoing I-131 therapy for DTC. While conventional multi-timepoint imaging remains the reference standard for comprehensive kinetic and dosimetric assessment, its routine clinical implementation is often limited by logistical and practical constraints. The proposed dual-timepoint protocol is therefore primarily intended as a complementary approach, with the potential to serve as a pragmatic substitute in selected clinical scenarios where simplified yet reproducible estimation of T_eff_ is sufficient.

## Methods

### Study design and ethics

This prospective observational study was conducted at the Kyoto University Hospital. The study protocol was approved by the institutional ethics committee (Approval number: R3598), and written informed consent was obtained from all participants. The study adhered to the principles of the Declaration of Helsinki and applicable Japanese ethical guidelines.

### Patient selection

Eligible participants included adult patients (≥ 18 years) with differentiated thyroid carcinoma who had undergone total thyroidectomy and were admitted for I-131 therapy. Patients were included if iodine-avid lesions were expected, defined as either (1) patients undergoing initial I-131 therapy with anticipated uptake in the thyroid bed, or (2) patients treated for metastatic disease with previously confirmed iodine-avid lesions on prior I-131 imaging.

**Fig. 1:**
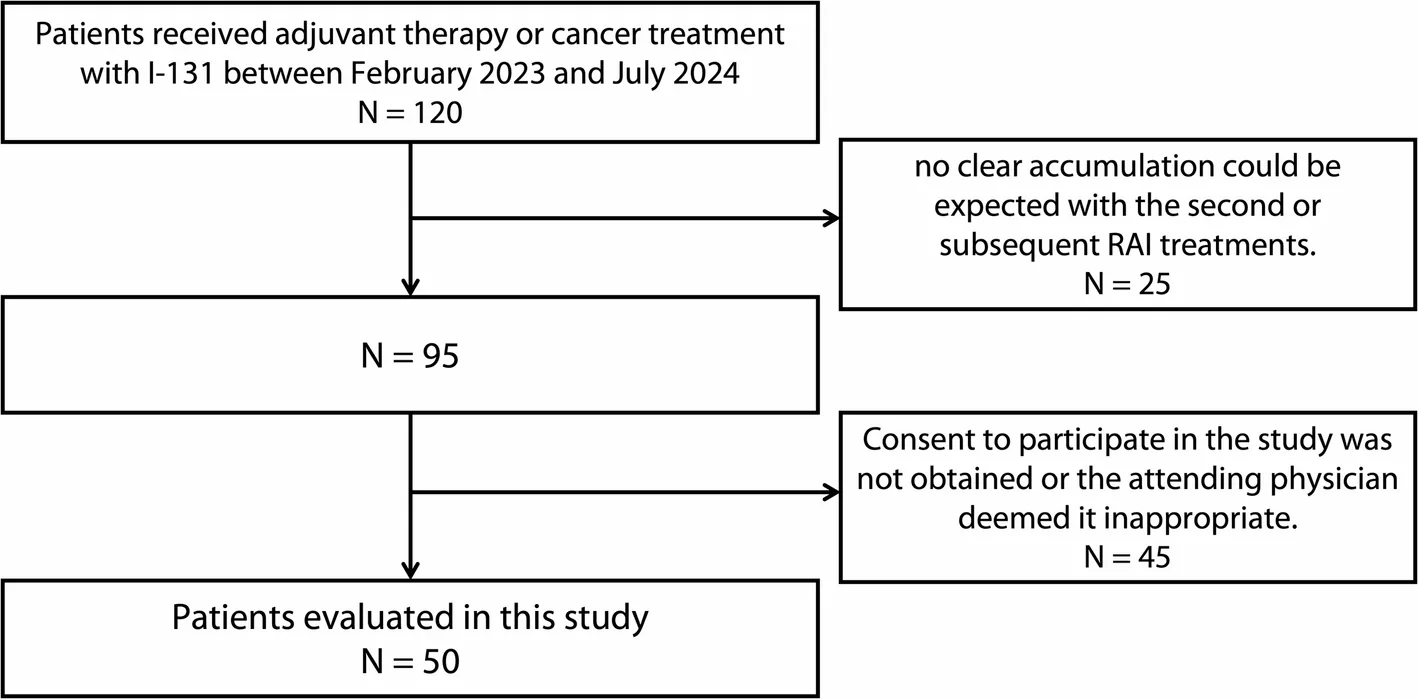
Flow diagram of patient selection. Between February 2023 and July 2024, 120 patients received adjuvant or therapeutic radioiodine (I-131) treatment. Among them, 25 patients were excluded because no clear iodine accumulation was expected during second or subsequent I-131 treatments. Of the remaining 95 patients, 45 were excluded due to lack of informed consent, or because the attending physician deemed study participation inappropriate. Ultimately, 50 patients were included in the final analysis

From February 2023 to July 2024, 120 patients were admitted for I-131 therapy. Among them, 57 patients had metastatic disease, of whom 25 were excluded due to the absence of expected iodine uptake. Additional patients were excluded as informed consent was not obtained, or the treating physician judged the patient to be unsuitable for study participation. Thus, a total of 50 patients were prospectively enrolled and included in the final analysis (Fig. 1). Because this study was designed as a prospective exploratory feasibility study, no formal sample size calculation was performed. Patients were consecutively enrolled during the study period, and the target sample size was determined based on the expected number of eligible patients undergoing I-131 therapy at our institution.

### I-131 administration and imaging protocol

Patients received a median therapeutic activity of 3700 MBq of I-131 (range, 3700–5550 MBq), administered orally. Whole-body planar scintigraphy was performed twice after administration: an early scan at 1.98–3.25 days, and delayed scan at 8.97–17.16 days. The interval between the two scans ranged from 6.18 to 14.28 (mean, 12.35) days. Exact administration and imaging times were retrospectively verified using the radiology information system to ensure accurate calculation of the time interval. The early and delayed imaging time points were selected based on institutional clinical practice and prior kinetic studies demonstrating that these intervals allow for the reliable estimation of T_eff_, while remaining feasible in routine clinical settings.

**Fig. 2:**
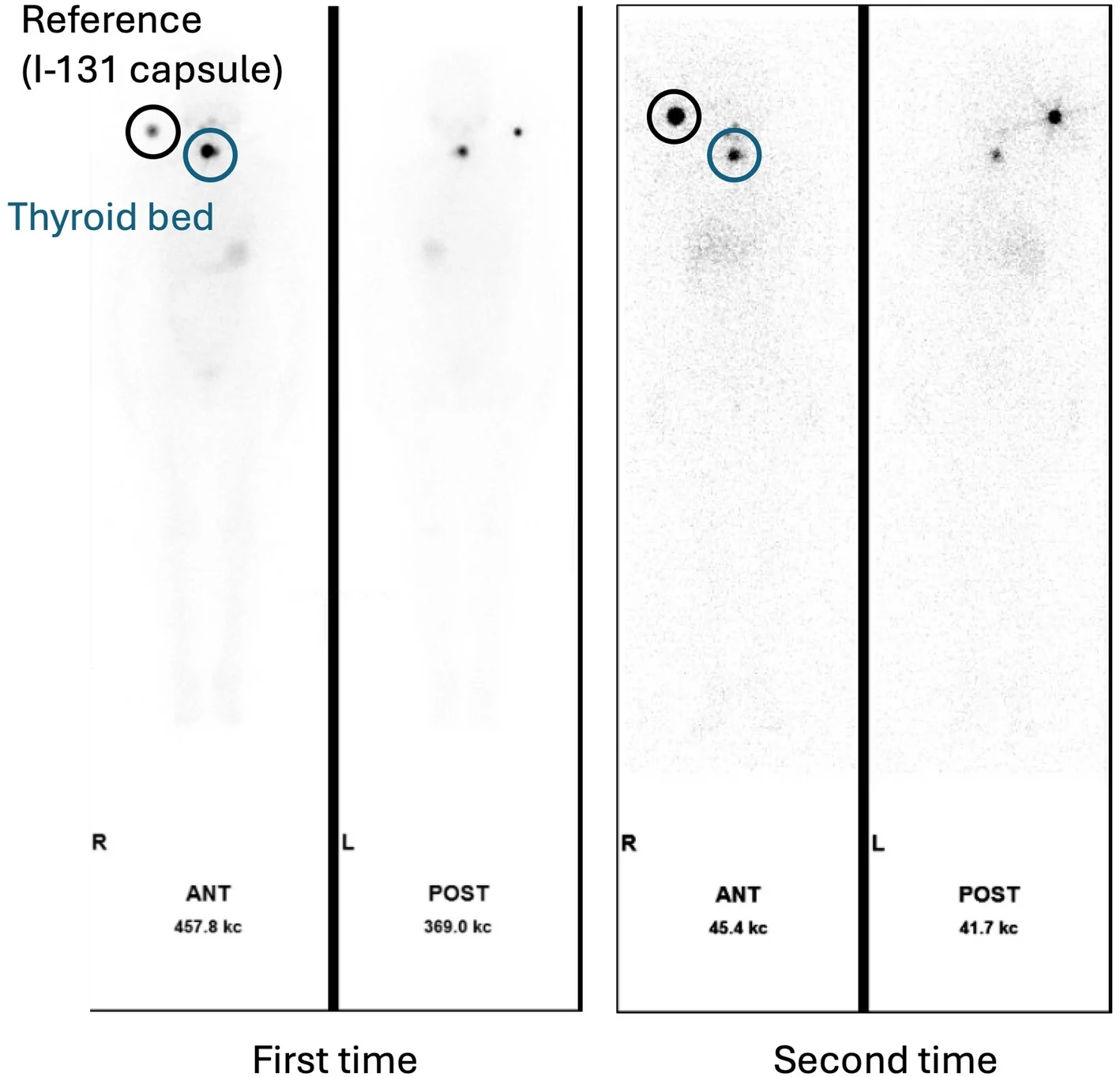
Whole-body planar scintigraphy acquired at discharge and follow-up after radioiodine (I-131) administration. Circular regions of interest (5 cm in diameter) were placed on planar images to quantify lesion activity. An internal reference capsule containing a known amount of I-131 activity was positioned near the head of the patient and used for session-specific activity calibration

During each scan, a sealed I-131 capsule with known activity (≤ 5 MBq) was placed near the head of the patient as an internal calibration reference. Additional radiation exposure from this capsule was minimal, estimated to be ≤ 0.13 mSv. Anterior and posterior whole-body planar images were acquired using a gamma camera equipped with high-energy collimators, using a 364 keV photopeak with a ± 10% energy window, a matrix size of 256 × 1024, and a scan speed of 15 cm/min (NM/CT 870 DR; GE Healthcare, Chicago, IL, USA). The arithmetic mean of anterior and posterior counts was employed, as this approach demonstrated superior quantitative stability for high-count I-131 imaging in our previous evaluation [7] (Fig. 2).

### Quantitative reproducibility assessment

Quantitative reproducibility of planar imaging was evaluated by comparing activity–count calibration slopes derived from a reference I-131 capsule between two independent imaging sessions.

During each imaging session, a linear regression was constructed between the known capsule activity and measured planar counts to obtain a session-specific calibration slope, representing the activity–count conversion factor. Reproducibility was assessed by calculating the percent difference between the calibration slopes obtained from the first and second imaging sessions. This slope difference served as an index of intersession stability of planar activity quantification.

The calibration slope obtained from each imaging session was subsequently applied to estimate the lesion activity within the same session.

### Region of interest analysis and T_eff_ estimation

Quantitative analysis of planar counts was performed using dedicated nuclear medicine image analysis software (MIM Maestro; MIM Software Inc., Cleveland, OH, USA). Regions of interest (ROIs) were defined using fixed-size circles with a diameter of 5 cm, which was projected onto anterior and posterior planar images as circular ROIs. For each lesion, the ROI was positioned such that the pixel with the maximum activity was located at the center of the ROI, ensuring consistent ROI placement across imaging sessions. The 5-cm diameter was selected to maintain consistency with our previous methodological validation study of I-131 planar quantification; in this pilot analysis, this ROI size showed favorable performance [7].

For each lesion, ROIs were independently placed on the anterior and posterior images, and the arithmetic mean of the measured counts was calculated and used as the representative lesion activity (Average) for quantitative analysis. This anterior–posterior averaging approach was adopted to reduce the effects of depth-dependent attenuation and improve quantitative robustness. Although the geometric mean is commonly used to reduce depth-dependent attenuation effects in planar quantification, we retained the arithmetic mean because our prior validation suggested greater stability for I-131 high-count planar imaging under our acquisition conditions. Although the inter- and intraobserver variability was not formally assessed, the use of a fixed-size, maximum-pixel–centered ROI protocol was intended to minimize observer-dependent variability. Only lesions demonstrating visually discernible iodine uptake and measurable activity at both imaging time points were included in the T_eff_ analysis.

Lesion-specific T_eff_ values were calculated from planar images acquired at two time points: the first at approximately 2.71 ± 0.19 days, and the second after an interval of 6.18–14.28 days (mean, 12.35 days) following I-131 administration. Lesion-specific T_eff_ was derived under the assumption that after the dominant uptake phase, lesion activity decreases according to a mono-exponential clearance model. In this model, lesion activity at time * t* is expressed as:

$$\:A\left(t\right)={A}_{0}{e}^{-{\lambda\:}_{\mathrm{e}\mathrm{f}\mathrm{f}}t}$$

where $\:{\lambda\:}_{\mathrm{e}\mathrm{f}\mathrm{f}}$ is the effective decay constant. Therefore, for two imaging time points separated by $\:{T}_{\mathrm{i}\mathrm{n}\mathrm{t}\mathrm{e}\mathrm{r}\mathrm{v}\mathrm{a}\mathrm{l}}$, the activity ratio is expressed as:

$$\:\frac{{A}_{2}}{{A}_{1}}={e}^{-{\lambda\:}_{\mathrm{e}\mathrm{f}\mathrm{f}}{T}_{\mathrm{i}\mathrm{n}\mathrm{t}\mathrm{e}\mathrm{r}\mathrm{v}\mathrm{a}\mathrm{l}}}$$

Accordingly, $\:{T}_{\mathrm{e}\mathrm{f}\mathrm{f}}$ was calculated as follows:

$$\:{T}_{\mathrm{e}\mathrm{f}\mathrm{f}}=\frac{{T}_{\mathrm{i}\mathrm{n}\mathrm{t}\mathrm{e}\mathrm{r}\mathrm{v}\mathrm{a}\mathrm{l}}\mathrm{l}\mathrm{n}2}{\mathrm{l}\mathrm{n}({A}_{1}/{A}_{2})}$$

where $\:{A}_{1}$ and $\:{A}_{2}$ represent the calibrated lesion activities at the first and second scans, respectively. Only lesions with $\:{A}_{2}<{A}_{1}$ were included in this calculation.

In patients with multiple iodine-avid lesions, the T_eff_ was evaluated separately for each lesion. Thyroid bed lesions and metastatic lesions were analyzed independently.

The biological half-life (T_bio_) was additionally calculated using the physical half-life of I-131 ($\:{T}_{\mathrm{p}\mathrm{h}\mathrm{y}\mathrm{s}}=8.02$ days) according to the following relationship:

$$\:\frac{1}{{T}_{\mathrm{e}\mathrm{f}\mathrm{f}}}=\frac{1}{{T}_{\mathrm{p}\mathrm{h}\mathrm{y}\mathrm{s}}}+\frac{1}{{T}_{\mathrm{b}\mathrm{i}\mathrm{o}}}$$

Therefore,

$$\:{T}_{\mathrm{b}\mathrm{i}\mathrm{o}}=\frac{{T}_{\mathrm{e}\mathrm{f}\mathrm{f}}\times\:{T}_{\mathrm{p}\mathrm{h}\mathrm{y}\mathrm{s}}}{{T}_{\mathrm{p}\mathrm{h}\mathrm{y}\mathrm{s}}-{T}_{\mathrm{e}\mathrm{f}\mathrm{f}}}$$

### Statistical analysis

Descriptive statistics are presented as means ± standard deviations or medians with interquartile ranges. Welch’s t-test was chosen to account for unequal variances between lesion groups. Given the exploratory nature of this study, no formal sample size calculation was performed. Statistical analyses were conducted using R software (version 4.3; R Foundation for Statistical Computing, Vienna, Austria), with* p*-values < 0.05 considered statistically significant.

## Results

### Patient and lesion characteristics

Among the 50 enrolled patients, the median age was 55 (range, 22–78) years, and 62% were female. Residual thyroid bed uptake was observed in 35 patients. Metastatic lesions were identified in 15 patients and included 11 bone, 5 lung, and 3 lymph node metastases. Several patients showed uptake in both the thyroid bed and metastatic sites. All patients successfully completed both imaging sessions, allowing for the calculation of T_eff_ in all enrolled cases.

### Planar imaging reproducibility

The activity–count calibration slopes derived from the internal reference I-131 capsule were 0.0941 and 0.0989 in the first and second imaging sessions, respectively, corresponding to a 5.0% intersession difference; this indicates a high intersession reproducibility of planar-based activity quantification.

### T_eff_ in thyroid bed and metastases

**Fig. 3:**
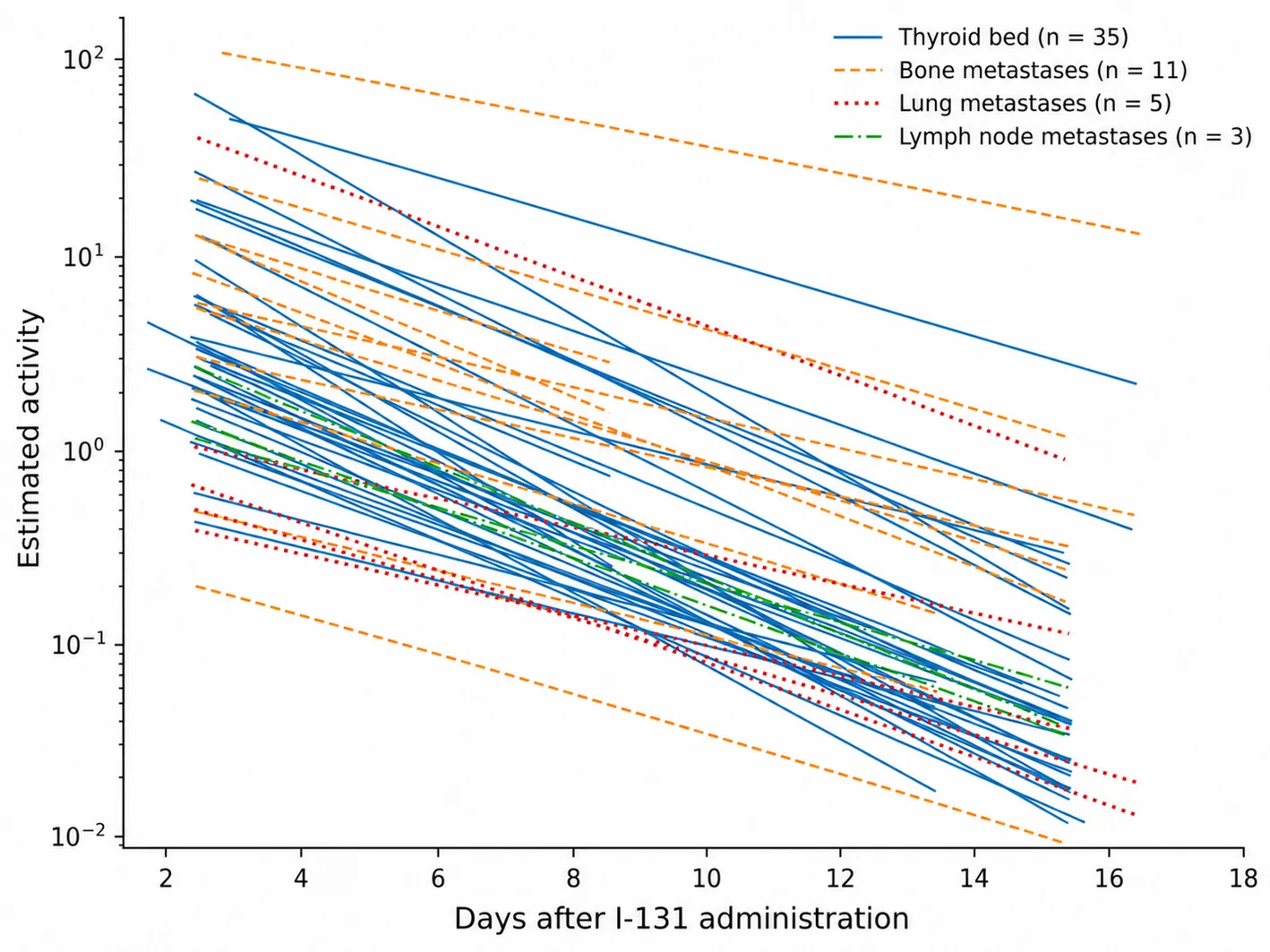
Dual-timepoint time–activity curves for individual lesions displayed on a logarithmic scale. Blue solid lines represent thyroid bed lesions (*n* = 35), orange dashed lines represent bone metastases (*n* = 11), red dotted lines represent lung metastases (*n* = 5), and green dash-dot lines represent lymph node metastases (*n* = 3). The thyroid bed lesions generally show a steeper activity decline, indicating shorter effective half-lives than metastatic lesions

For thyroid bed lesions (*n* = 35), the mean T_eff_ was 2.16 ± 0.56 days, with a range of 1.34 to 3.61 days. Among metastatic lesions demonstrating sufficient iodine uptake and complete dual-timepoint data, bone metastases (*n* = 11 lesions) exhibited a mean T_eff_ of 3.22 ± 0.76 (range, 2.07–4.56) days, and lung metastases (*n* = 5 lesions) had a mean T_eff_ of 3.36 ± 0.80 (range, 2.42–4.16) days (Fig. 3). Owing to the small number of metastatic lesions, particularly lung metastases, no formal statistical comparison was performed between metastatic subgroups. Welch’s t-test demonstrated that T_eff_ in the thyroid bed was significantly shorter than in metastatic lesions (*p* < 0.001). The corresponding mean T_bio_ was 3.08 ± 1.23 days for thyroid bed lesions, 5.72 ± 2.36 days for bone metastases, and 5.64 ± 2.33 days for lung metastases.

## Discussion

This study evaluated the feasibility and clinical applicability of dual-timepoint planar imaging with internal capsule calibration for estimating lesion-specific T_eff_ in patients undergoing I-131 therapy for DTC. The first objective was to assess quantitative reproducibility, as reflected by the stability of the calibration slope derived from an internal reference I-131 capsule. The observed intersession slope difference of approximately 5.0% indicates that the activity–count relationship remained highly stable across imaging sessions.

Because lesion activity estimation and subsequent T_eff_ calculation were performed using session-specific calibration slopes, this level of reproducibility is sufficient to support reliable lesion-specific dosimetry in routine clinical practice. The use of an internal reference capsule enables calibration under identical acquisition conditions for each imaging session, thereby minimizing system- and time-dependent variability.

The second objective focused on characterizing lesion-specific T_eff_ in the thyroid bed and metastatic lesions. Our results demonstrated significant differences between lesion types, with thyroid bed lesions exhibiting shorter T_eff_ than metastatic lesions. The shorter T_eff_ observed in the thyroid bed likely reflects more rapid biological clearance from remnant thyroid tissue, whereas metastatic lesions—including bone and lung metastases—tended to exhibit prolonged iodine retention. Such heterogeneity in iodine kinetics among lesion types has been previously reported and underscores the importance of lesion-specific kinetic assessment [5, 8–10]. Previous studies, including the study by Klain et al. [11], have demonstrated substantial variability in whole-body I-131 T_eff_ among patients with DTC. The present study extends this broader kinetic framework by focusing on lesion-specific T_eff_, showing differences between thyroid bed remnants and metastatic lesions. The additionally calculated T_bio_ values showed a similar lesion-type-dependent pattern to T_eff_, supporting the presence of differences in biological iodine retention between thyroid bed remnants and metastatic lesions.

Accurate characterization of lesion-specific iodine kinetics is a key prerequisite for individualized dosimetry in I-131 therapy, as absorbed dose is directly influenced by T_eff_ [4, 5]. Several studies have demonstrated that lesion-specific dosimetric parameters may substantially deviate from assumptions underlying empiric dosing strategies, supporting more personalized treatment planning approaches [8, 12]. From a clinical perspective, these differences in lesion-specific T_eff_ may translate into substantial variability in absorbed dose when empiric activity prescriptions are applied, highlighting the potential value of simplified kinetic assessment even when full multi-timepoint dosimetry is not feasible. Time-integrated activity is more directly related to absorbed dose than T_eff_. Under the mono-exponential postuptake clearance assumption, TIA after the first imaging time point could be approximated from the measured activity and effective decay constant. However, total TIA over the complete time–activity curve depends on clearance, as well as uptake phase and peak activity, which were not directly characterized in the present dual-timepoint protocol. Therefore, the present study focused on lesion-specific T_eff_ and T_bio_ as simplified kinetic parameters; future studies incorporating earlier and multiple time points are warranted for robust TIA and absorbed-dose estimation.

The simplified dual-timepoint imaging protocol used in this study substantially reduces patient burden and logistical complexity compared with conventional multi-timepoint dosimetry protocols [6, 13]. This approach relies on the assumption that after the dominant uptake or redistribution phase, lesion activity decreases in a predominantly mono-exponential manner. Under this condition, two appropriately separated imaging time points can be used to approximate T_eff_ from the log-linear decline in activity. Previous studies in molecular radiotherapy have demonstrated that simplified two-timepoint protocols can reduce imaging burden while providing clinically useful kinetic or dosimetric estimates under appropriate modeling assumptions [7, 13–15]. Importantly, recent work on two-timepoint dosimetry has also emphasized that tumor TIA estimation depends on both uptake and washout phases, supporting the need for cautious interpretation when early uptake data are omitted [15]. Therefore, the proposed method should be interpreted as a practical approximation of postuptake clearance kinetics, rather than a full characterization of the complete time–activity curve.

Planar imaging was intentionally selected to prioritize quantitative stability and reproducibility. Although SPECT/CT offers theoretical advantages for three-dimensional quantification, quantitative accuracy in I-131 imaging may be affected by reconstruction parameters and high-energy photon penetration [16–18]. In our previous comparison of planar and SPECT imaging for I-131 scintigraphy, planar imaging demonstrated superior correlation with known activity and reduced susceptibility to reconstruction-related artifacts [7]. The present study extends these findings by demonstrating sufficient temporal stability of planar imaging for T_eff_ estimation in a clinical setting, supporting its use in routine practice where robustness and simplicity are prioritized.

An additional practical insight from this study is the importance of precise temporal information. Verification of exact administration and imaging times using the radiology information system revealed minor discrepancies, and correction of these discrepancies improved the consistency of calibration slopes. This finding highlights that accurate time management is a critical factor for reliable estimation of T_eff_, particularly when simplified kinetic models are applied [6, 13].

Certain limitations should be acknowledged; first, the dual-timepoint method assumes that lesion activity after the dominant uptake phase can be approximated by mono-exponential clearance, and therefore does not capture the full time–activity curve. This assumption may not hold in lesions with delayed uptake, prolonged redistribution, heterogeneous iodine retention, or complex biphasic kinetics. In such cases, omission of the early uptake phase may lead to biased T_eff_ estimates, and multi-timepoint imaging remains preferable for comprehensive kinetic characterization. The timing of the first scan is another important consideration. In the present protocol, the early scan was acquired at a mean of 2.71 days after I-131 administration. Although this timing was selected to fit routine clinical workflow and obtain sufficient count statistics, some lesions—particularly thyroid bed remnants—may still be undergoing uptake or redistribution at this time. If the first scan is acquired before the clearance phase is fully established, the mono-exponential assumption may be violated, potentially leading to biased T_eff_ estimates. A later first imaging time point, such as day 4–5, might better represent the postuptake clearance phase in selected cases; however, this approach would need to be balanced against reduced count statistics, patient burden, and logistical feasibility. Variability in the delayed imaging timepoint may also influence T_eff_ estimation. In dual-timepoint analysis, the precision of T_eff_ depends on both the accuracy of the activity measurements and the temporal separation between the two scans. Shorter intervals may amplify the effect of count uncertainty on the estimated slope of the log-linear activity decline, whereas excessively delayed imaging may be affected by low residual lesion counts and reduced detectability. Although the exact imaging times were incorporated into the T_eff_ calculation in the present study, a dedicated simulation study or comparison with multi-timepoint reference data would be useful to optimize the timing of image acquisition and quantify timing-related uncertainty.

In addition, the relatively small number of metastatic lesions available for subgroup analysis, particularly lung metastases, limits the strength of conclusions regarding differences among metastatic sites and warrants cautious interpretation. In particular, the numerically similar T_eff_ values observed for bone and lung metastases should be interpreted descriptively, as the study was not powered to demonstrate equivalence or detect small differences between metastatic subgroups. Nevertheless, previous reports and the present data suggest that deviations in T_eff_ and absorbed dose estimation introduced by this simplification generally remain within approximately 10%, which is acceptable for routine clinical dosimetry when balanced against feasibility and patient burden [14, 19]. Additionally, the use of a fixed 5-cm circular ROI may have introduced lesion-size-dependent effects, including partial-volume effects in small lesions and background contamination in heterogeneous thyroid bed remnants or adjacent physiologic uptake. Although this ROI size was selected based on our previous validation work, and a predefined maximum-pixel-centered placement protocol was used to reduce observer-dependent variability, inter- and intraobserver variabilities were not formally assessed in the present study. Therefore, the potential influence of ROI placement and lesion size on T_eff_ estimation should be acknowledged, and future studies should evaluate ROI-size sensitivity and observer reproducibility in larger lesion-based datasets.

Overall, dual-timepoint planar imaging with internal capsule calibration provides a practical, physically robust, and reproducible method for estimating lesion-specific T_eff_ in I-131 therapy for DTC. This approach offers an appropriate balance between quantitative accuracy and clinical feasibility and may serve as a useful foundation for individualized dosimetry in daily clinical practice, particularly in settings where comprehensive multi-timepoint imaging is impractical.

## Conclusions

Dual-timepoint planar imaging with internal I-131 capsule calibration enables reproducible estimation of lesion-specific T_eff_ in patients undergoing I-131 therapy for DTC. This approach provides a practical balance between quantitative accuracy and clinical feasibility, allowing reliable characterization of iodine kinetics with minimal imaging burden. The observed differences in T_eff_ between thyroid bed and metastatic lesions highlight the importance of lesion-specific kinetic assessment and support the potential role of this method as a foundation for individualized dosimetry in routine clinical practice.

## Data Availability

The datasets generated and/or analyzed during the current study are not publicly available due to institutional and ethical restrictions but are available from the corresponding author on reasonable request.

## Abbreviations

T_eff_: Effective half-life
T_bio_: Biological half-life
DTC: Differentiated thyroid cancer
SPECT: Single-photon emission computed tomography
CT: Computed tomography
ROI: Region of interest

## References

[1] Vigneri R, Malandrino P, Vigneri P. The changing epidemiology of thyroid cancer: why is incidence increasing? Curr Opin Oncol. 2015;27:1–7. 10.1097/CCO.0000000000000148. 25310641

[2] Hori M, Matsuda T, Shibata A, Katanoda K, Sobue T, Nishimoto H, et al. Cancer incidence and incidence rates in Japan in 2009: a study of 32 population-based cancer registries for the Monitoring of Cancer Incidence in Japan (MCIJ) project. Jpn J Clin Oncol. 2015;45:884–91. 10.1093/jjco/hyv088. 26142437

[3] Haugen BR, Alexander EK, Bible KC, Doherty GM, Mandel SJ, Nikiforov YE, et al. 2015 American Thyroid Association management guidelines for adult patients with thyroid nodules and differentiated thyroid cancer: the American thyroid association guidelines task force on thyroid nodules and differentiated thyroid cancer. Thyroid. 2016;26:1–133. 10.1089/thy.2015.0020. 26462967 PMC4739132

[4] Hänscheid H, Canzi C, Eschner W, Flux G, Luster M, Strigari L, et al. EANM dosimetry committee series on standard operational procedures for pre-therapeutic dosimetry II. Dosimetry prior to radioiodine therapy of benign thyroid diseases. Eur J Nucl Med Mol Imaging. 2013;40:1126–34. 10.1007/s00259-013-2387-x. 23576099

[5] Robbins RJ, Schlumberger MJ. The evolving role of (131)I for the treatment of differentiated thyroid carcinoma. J Nucl Med. 2005;46(Suppl 1):S28–37. 15653649

[6] Lassmann M, Chiesa C, Flux G, Bardiès M. EANM dosimetry committee guidance document: good practice of clinical dosimetry reporting. Eur J Nucl Med Mol Imaging. 2011;38:192–200. 10.1007/s00259-010-1549-3. 20799035

[7] Iizuka Y, Katagiri T, Inoue M, Nakamura K, Mizowaki T. Comparison between planar and single-photon computed tomography images for radiation intensity quantification in iodine-131 scintigraphy. Sci Rep. 2021;11:21858. 10.1038/s41598-021-01432-x. 34750482 PMC8576011

[8] Maxon HR 3rd, Englaro EE, Thomas SR, Hertzberg VS, Hinnefeld JD, Chen LS, et al. Radioiodine-131 therapy for well-differentiated thyroid cancer—a quantitative radiation dosimetric approach: outcome and validation in 85 patients. J Nucl Med. 1992;33:1132–6. 1597728

[9] Dorn R, Kopp J, Vogt H, Heidenreich P, Carroll RG, Gulec SA. Dosimetry-guided radioactive iodine treatment in patients with metastatic differentiated thyroid cancer: largest safe dose using a risk-adapted approach. J Nucl Med. 2003;44:451–6. 12621014

[10] Tuttle RM, Leboeuf R, Robbins RJ, Qualey R, Pentlow K, Larson SM, et al. Empiric radioactive iodine dosing regimens frequently exceed maximum tolerated activity levels in elderly patients with thyroid cancer. J Nucl Med. 2006;47:1587–91. 17015892

[11] Klain M, Nappi C, De Risi M, Piscopo L, Volpe F, Manganelli M, et al. Whole-body radioiodine effective half-life in patients with differentiated thyroid cancer. Diagnostics. 2021;11:1740. 10.3390/diagnostics11101740. 34679438 PMC8535104

[12] Sgouros G, Kolbert KS, Sheikh A, Pentlow KS, Mun EF, Barth A, et al. Patient-specific dosimetry for 131I thyroid cancer therapy using 124I PET and 3-dimensional-internal dosimetry (3D-ID) software. J Nucl Med. 2004;45:1366–72. 15299063

[13] Gear JI, Cox MG, Gustafsson J, Gleisner KS, Murray I, Glatting G, et al. EANM practical guidance on uncertainty analysis for molecular radiotherapy absorbed dose calculations. Eur J Nucl Med Mol Imaging. 2018;45:2456–74. 10.1007/s00259-018-4136-7. 30218316 PMC6208822

[14] Flux G, Bardies M, Monsieurs M, Savolainen S, Strand SE, Lassmann M, EANM Dosimetry Committee. The impact of PET and SPECT imaging on dosimetry for targeted radionuclide therapy. Z Med Phys. 2006;16:47–59. 10.1078/0939-3889-00291. 16696370

[15] Chen G, Lu Z, Jiang H, Afshar-Oromieh A, Rominger A, Shi K, et al. Lu-177-PSMA dosimetry for kidneys and tumors based on SPECT images at two imaging time points. Front Med (Lausanne). 2023;10:1246881. 10.3389/fmed.2023.1246881. 38020081 PMC10679348

[16] Dickson J, Ross J, Vöö S. Quantitative SPECT: the time is now. EJNMMI Phys. 2019;6:4. 10.1186/s40658-019-0241-3. 30830530 PMC6399365

[17] Dewaraja YK, Frey EC, Sgouros G, Brill AB, Roberson P, Zanzonico PB, et al. MIRD pamphlet 23: quantitative SPECT for patient-specific 3-dimensional dosimetry in internal radionuclide therapy. J Nucl Med. 2012;53:1310–25. 10.2967/jnumed.111.100123. 22743252 PMC3465844

[18] Ljungberg M, Pretorius PH. SPECT/CT: an update on technological developments and clinical applications. Br J Radiol. 2018;91:20160402. 10.1259/bjr.20160402. 27845567 PMC5966195

[19] Ljungberg M, Sjögreen Gleisner K. Personalized dosimetry for radionuclide therapy using molecular imaging tools. Biomedicines. 2016;4:25. 10.3390/biomedicines4040025. 28536392 PMC5344265

